# Accuracy of Self-reported Human Papillomavirus Vaccination Status Among Gay and Bisexual Adolescent Males: Cross-sectional Study

**DOI:** 10.2196/32407

**Published:** 2021-12-06

**Authors:** Eric PF Chow, Christopher K Fairley, Rebecca Wigan, Jane S Hocking, Suzanne M Garland, Alyssa M Cornall, Sepehr N Tabrizi, Marcus Y Chen

**Affiliations:** 1 Melbourne Sexual Health Centre Alfred Health Melbourne Australia; 2 Central Clinical School Faculty of Medicine, Nursing and Health Sciences Monash University Melbourne Australia; 3 Centre for Epidemiology and Biostatistics Melbourne School of Population and Global Health The University of Melbourne Melbourne Australia; 4 Department of Obstetrics and Gynaecology Melbourne Medical School The University of Melbourne Melbourne Australia; 5 Murdoch Children’s Research Institute Melbourne Australia; 6 Centre for Women’s Infectious Diseases The Royal Women's Hospital Melbourne Australia

**Keywords:** human papillomavirus, vaccination, accuracy, self-reported, men who have sex with men, immunisation, public health, immunization, HPV vaccination, bisexual adolescents, bisexual men

## Abstract

**Background:**

Men who have sex with men are a risk group for anal human papillomavirus (HPV) and anal cancer. Australia introduced a universal school-based HPV vaccination program in 2013. Self-reported HPV vaccination status has been widely used in clinical and research settings, but its accuracy is understudied.

**Objective:**

We aimed to examine the accuracy of self-reported HPV vaccination status among gay and bisexual adolescent males.

**Methods:**

We included 192 gay and bisexual males aged 16-20 years from the Human Papillomavirus in Young People Epidemiological Research 2 (HYPER2) study in Melbourne, Australia. All participants had been eligible for the universal school-based HPV vaccination program implemented in 2013 and were asked to self-report their HPV vaccination status. Written informed consent was obtained to verify their HPV vaccination status using records at the National HPV Vaccination Program Register and the Australian Immunisation Register. We calculated the sensitivity, specificity, positive predictive value, and negative predictive value of self-reported HPV vaccination status.

**Results:**

The median age of the 192 males was 19 (IQR 18-20) years. There were 128 males (67%) who had HPV vaccination records documented on either registry. Self-reported HPV vaccination had a sensitivity of 47.7% (95% CI 38.8%-56.7%; 61/128), a specificity of 85.9% (95% CI 75.0%-93.4%; 55/64), a positive predictive value of 87.1% (95% CI 77.0%-93.9%; 61/70), and a negative predictive value of 45.1% (95% CI 36.1%-54.3%; 55/122).

**Conclusions:**

Self-reported HPV vaccination status among Australian gay and bisexual adolescent males underestimates actual vaccination and may be inaccurate for clinical and research purposes.

## Introduction

Multiple countries have implemented national human papillomavirus (HPV) vaccination programs and demonstrated significant reductions in HPV infection, genital warts, and cervical cancer and its precursors [1-4]. Self-reported HPV vaccination status has been widely used in clinical and research settings to monitor the effectiveness of the HPV vaccination program.

Australia introduced a school-based HPV vaccination program for girls in 2007, with expansion to include 12- and 13-year-old boys in 2013. Additionally, Australia established robust population-based vaccination registries to document HPV vaccination coverage [5]. Several studies have examined the accuracy of self-reported HPV vaccination status among women and adolescent girls; however, the proportion who report their HPV vaccination status correctly varies across populations and settings, ranging from 54% to 92% [6-15]. Low sensitivity (54%) of self-reported HPV vaccination status against medical records is reported among adolescent girls aged 14-17 years in the United States [6]. The sensitivity seems to be improved among older populations. Another US study has shown that the sensitivity of self-reported HPV vaccination status against medical records is 91% among women aged 18-49 years [10]. A reasonable but lower sensitivity of self-reported HPV vaccination status is also reported when immunization registry data are used as the reference standard, with a sensitivity of 85% among women aged 20-22 years in Japan and 86% among women aged 22-30 years in Australia [7,12].

Most previous studies verified self-reported HPV vaccination status from parents or electronic medical records instead of vaccination registries. One study examined the accuracy of self-reported HPV vaccination status in men, but the authors did not separate the data of heterosexual men and gay/bisexual men [16]. Australia is one of very few countries that has implemented a school-based HPV vaccination program coupled with a national vaccination registry recording HPV vaccination. This study aimed to determine the accuracy of self-reported HPV vaccination status from the school-based program verified against the national vaccination registry among gay and bisexual adolescent males.

## Methods

The Human Papillomavirus in Young People Epidemiological Research 2 (HYPER2) study was a cross-sectional study aimed at examining HPV prevalence among young gay and bisexual males after the implementation of the school-based gender-neutral HPV vaccination program in Australia, with the main findings published elsewhere [17]. A total of 200 same-sex attracted men aged 16-20 years who were residents in Australia in 2013 were recruited via the HYPER2 study to ensure they had been eligible for the gender-neutral HPV vaccination program at the time. All males were recruited at the Melbourne Sexual Health Centre (Victoria, Australia) between January 2017 and March 2019. All men were asked to complete a questionnaire that collected demographic characteristics, sexual practices, and self-reported HPV vaccination status. Men could choose “unsure” for vaccination status and the number of doses. Written consent was obtained from all the men. Ethics approval was granted from the Alfred Hospital Ethics Committee, Melbourne, Australia (429/16).

The National HPV Vaccination Program Register (NHVPR) was established in 2008 to monitor HPV vaccination coverage in Australia. State Health Departments and local councils were mandated to report individuals who received the vaccine from the school-based program to the NHVPR [5]. The NHVPR also received vaccination records on a voluntary basis from general practices (who received notification payments for doing so in the initial 3 years when mass catch-up through general practice occurred) and other immunization providers across Australia. The NHVPR ceased at the end of 2018 and HPV vaccination records moved to the Australian Immunization Register (AIR); therefore, NHVPR and AIR were both used to verify HPV vaccination records [18]. A probabilistic matching based on identifying details (eg, first and last name, date of birth) was used to identify an individual’s corresponding vaccination records in the registers.

Only men who received the vaccine via the school-based program were eligible for inclusion in this study. Self-reported HPV vaccination status was stratified into “vaccinated” and “not vaccinated or unsure” based on self-reported data on the survey. The sensitivity, specificity, positive predictive value, negative predictive value, positive likelihood ratio, and negative likelihood ratio for self-reported HPV vaccination status were calculated using data from the registers as the reference standard. The positive likelihood ratio is the probability of individuals who self-reported being vaccinated in those who were vaccinated divided by the probability of individuals who self-reported being vaccinated in those who were not vaccinated (ie, dividing the sensitivity by 1 minus the specificity). The negative likelihood ratio is the probability of individuals who self-reported being not vaccinated in those who were vaccinated divided by the probability of individuals who self-reported being not vaccinated in those who were not vaccinated (ie, dividing 1 minus the sensitivity by the specificity). A positive likelihood ratio >10 is useful for ruling in being vaccinated, while a negative likelihood ratio <0.1 is useful for ruling out being vaccinated [19,20]. The κ statistics were also calculated to determine the agreement between self-reported vaccination status and registry records. The level of agreement was categorized based on the κ statistics as none (0-0.20), minimal (0.21-0.39), weak (0.40-0.59), moderate (0.60-0.79), strong (0.80-0.90), and almost perfect (>0.90) [21]. We also performed analyses by excluding individuals who were unsure about their vaccination status. All statistical analyses were performed in Stata (version 17; StataCorp LLC).

## Results

Of the 200 men, 8 were excluded from the analysis because they were not vaccinated as part of the school-based vaccination program. Median age of the 192 men was 19 (IQR 18-20) years. Most men completed secondary school (n=138, 71.9%). The median number of lifetime male sex partners was 9 (IQR 5-25). There were 70 (36.5%) men who self-reported being vaccinated, 13 (6.8%) reported being unvaccinated, and 109 (56.8%) men were unsure about their vaccination status (Table 1).

**Table 1:** The 2×2 tables comparing self-reported vaccination status to the reference standard of national vaccine registry vaccination data among gay and bisexual adolescent males.

Self-reported vaccination status		Vaccination registries				Total
		Vaccinated		Not vaccinated		
**All men (N=192)**						
	Self-reported vaccinated	61	9		70	
	Self-reported not vaccinated/unsure	67	55		122	
	Total	128	64		192	
**All men excluding those who were unsure about their vaccination (N=83)^a^**						
	Self-reported vaccinated	61	9		70	
	Self-reported not vaccinated	1	12		13	
	Total	62	21		83	

^a^This value excludes the 109 men who were unsure about their vaccination status.

At least one dose of HPV vaccination was recorded in the HPV vaccine registry for 66.7% (128/192) of men, 63.0% (121/192) completed 3 doses of vaccination, 6 men received 2 doses, and 1 man received 1 dose. Only 61 of the 128 men correctly reported they were vaccinated (sensitivity=47.7%; Table 2). Of those 64 men who did not have any registry record, 55 men reported being unvaccinated or unsure of their vaccination status (specificity=85.9%). The positive predictive value was 87.1% (61/70) and the negative predictive value was 45.1% (55/122). The positive likelihood ratio was 3.4 and the negative likelihood ratio was 0.61.

**Table 2:** Accuracy of self-reported human papillomavirus vaccination status among gay and bisexual adolescent males using national vaccine registry data as the reference standard.

Diagnostic accuracy	All men (N=192), value (95% CI)	All men excluding those who were unsure about their vaccination (N=83)^a^, value (95% CI)
Sensitivity, %	47.7 (38.8-56.7)	98.4 (91.3-100)
Specificity, %	85.9 (75.0-93.4)	57.1 (34.0-78.2)
Positive predictive value, %	87.1 (77.0-93.9)	87.1 (77-93.9)
Negative predictive value, %	45.1 (36.1-54.3)	92.3 (64.0-99.8)
Positive likelihood ratio	3.4 (1.8-6.4)	2.3 (1.4-3.8)
Negative likelihood ratio	0.61 (0.50-0.74)	0.03 (0-0.20)
κ value	0.274 (0.166-0.382)	0.635 (0.435-0.836)

^a^This value excludes the 109 men who were unsure about their vaccination status.

After excluding 109 men who were unsure about their vaccination status, there was an improvement in the sensitivity (61/62, 98.4%) and negative predictive value (12/13, 92.3%), but a decrease in the specificity (12/21, 57.1%). The positive likelihood ratio remained similar (2.3) but with a relatively low negative likelihood ratio (0.03).

The agreement between self-reported vaccination status and registry record was minimal (κ=0.274) when including men with unsure vaccination status in the unvaccinated group. However, the level of agreement between self-reported vaccination status and registry record improved to moderate (κ=0.635) when excluding men with unsure vaccination status. 

## Discussion

This study examines the accuracy of self-reported HPV vaccination status among gay and bisexual adolescent males using national vaccine registry data as the reference standard. Our study showed that, of those vaccinated with at least one dose, only 48% of men correctly recalled their vaccination status, with over half of men unsure. However, the sensitivity improved to 98% after excluding men who were unsure about their vaccination status.

The low sensitivity of self-reported vaccination status in our study is similar to a US study showing only 54% of 74 adolescent girls aged 14-17 years correctly reported their HPV vaccination status as verified via medical records [6]. To our best knowledge, there has been only one study examining the accuracy of self-reported HPV vaccination status among men. Consistent with our findings, a US study has also reported a minimal agreement (κ=0.35) between self-reported vaccination status and medical records among men aged 13-26 years [16]. The authors also reported a positive predictive value of 62% [16], which is lower than our estimate (87%); this is likely due to the United States having lower vaccination coverage compared to Australia. However, the authors did not report the sensitivity and specificity for heterosexual men and gay/bisexual men separately. Past studies have reported that the sensitivity of self-reported HPV vaccination status is higher among young adults compared to adolescents [16], and this may be because adolescents may receive multiple vaccines at school around the same time and they may not remember which specific vaccine they received. Several countries have also implemented catch-up HPV vaccination programs for gay and bisexual men aged up to 45 years [22-24]. Further studies examining the accuracy of self-reported HPV vaccination status among gay and bisexual men in these populations would be beneficial.

We found that the positive likelihood ratios were relatively small regardless of whether the unsure vaccination group was included or not. Given the positive likelihood ratios are <10 [19], this suggests that self-reported vaccination status is not useful for ruling in being vaccinated. However, there was a significant change in the negative likelihood ratio from 0.61 when including men who were unsure about their vaccination status to 0.03 when excluding men who were unsure about their vaccination status. A negative likelihood ratio that is <0.1 would be useful for ruling out being vaccinated [20]. Given not all individuals would be aware of their vaccination status, the high negative likelihood ratio in our study suggests that self-reported vaccination status is not useful for ruling out being vaccinated.

There are several limitations to this study. First, this study was conducted among adolescent gay and bisexual men; therefore, our findings may not be generalizable to other populations such as heterosexual men and adults. Second, adolescents also receive the meningococcal ACWY and whooping cough booster vaccines at school around the age they receive the HPV vaccine [25], and it is likely many adolescents cannot distinguish between the vaccines. Third, the vaccines given in general practice may not have been recorded in the registers and therefore we may have underestimated the proportion of vaccinated individuals in this population.

In conclusion, the accuracy of self-reported HPV vaccination status among gay and bisexual adolescent males was low, and most men were unsure about their vaccination status. Underreporting HPV vaccination suggests that self-reported vaccination status may be inaccurate for clinical practice to guide vaccination and for research evaluating the effectiveness of vaccination programs. This highlights the benefit of using data on actual vaccination status from vaccination registries to verify vaccination status and dosage. The AIR is the national register that records vaccines that are given to individuals in Australia. Individuals can access their immunization history statement online. Additionally, general practitioners or other vaccination providers can also access the immunization history statement online.

## Abbreviations

AIR: Australian Immunization Register
HPV: human papillomavirus
HYPER2: Human Papillomavirus in Young People Epidemiological Research 2
NHVPR: National HPV Vaccination Program Register
